# Cloning and Characterization of a Putative *TAC1* Ortholog Associated with Leaf Angle in Maize (*Zea mays* L.)

**DOI:** 10.1371/journal.pone.0020621

**Published:** 2011-06-07

**Authors:** Lixia Ku, Xiaomin Wei, Shaofang Zhang, Jun Zhang, Shulei Guo, Yanhui Chen

**Affiliations:** 1 College of Agronomy and Key Laboratory of Physiological Ecology and Genetic Improvement of Food Crops in Henan Province, Henan Agricultural University, Zhengzhou, China; 2 Zhengzhou College of Animal Husbandry Engineering, Zhengzhou, China; Tulane University Health Sciences Center, United States of America

## Abstract

**Background:**

Modifying plant architecture to increase photosynthesis efficiency and reduce shade avoidance response is very important for further yield improvement when crops are grown in high density. Identification of alleles controlling leaf angle in maize is needed to provide insight into molecular mechanism of leaf development and achieving ideal plant architecture to improve grain yield.

**Methodology/Principal Findings:**

The gene cloning was done by using comparative genomics, and then performing real-time polymerase chain reaction (RT-PCR) analysis to assay gene expression. The gene function was validated by sequence dissimilarity analysis and QTL mapping using a functional cleaved amplified polymorphism (CAP).

**Conclusions:**

The leaf angle is controlled by a major quantitative trait locus, *ZmTAC1* (*Zea mays* L. Leaf Angle Control 1). *ZmTAC1* has 4 exons encoding a protein with 263 amino acids, and its domains are the same as those of the rice *OsTAC1* protein. *ZmTAC1* was found to be located in the region of qLA2 by using the CAP marker and the F_2:3_ families from the cross between Yu82 and Shen137. Real-time PCR analysis revealed *ZmTAC1* expression was the highest in the leaf-sheath pulvinus, less in the leaf and shoot apical meristem, and the lowest in the root. A nucleotide difference in the 5′-untranslated region (UTR) between the compact inbred line Yu82 (“CTCC”) and the expanded inbred line Shen137 (“CCCC”) influences the expression level of *ZmTAC1*, further controlling the size of the leaf angle. Sequence verification of the change in the 5′-UTR revealed *ZmTAC1* with “CTCC” was present in 13 compact inbred lines and *ZmTAC1* with “CCCC” was present in 18 expanded inbred lines, indicating *ZmTAC1* had been extensively utilized in breeding with regard to the improvement of the maize plant architecture.

## Introduction

Change in maize leaf angle (LA) alters plant architecture. A plant with narrower leaf angles above uppermost ear is considered as compact plant architecture, while a plant with wider leaf angles above uppermost ear is considered as expanded plant architecture. Therefore, LA is one of the important factors that directly influence the yield by affecting the planting density of maize. For maize breeders, improving the yield remains an important objective of most current breeding programs. The canopy interception rate of light is a major restriction factor for improving the yield, and high yields can be obtained through more dense plantings. An important determinant influencing the canopy interception rate of light in maize is the spatial and temporal changes in the LA, which affects the plant's ability to survive, capture light and reproduce successfully.

Leaf formation occurs at the shoot apical meristem (SAM). The meristem is divided into the central zone where a group of slowly dividing stem cells is responsible for the self-maintenance of the meristem and the peripheral zone where leaf primordia are formed [1]. Surgical separation of incipient primordia from the meristem leads to radially symmetrical leaves, indicating that a signal from the meristem is required to establish dorsiventrality in leaves [2], [3]. The signal induces dorsal identity in the adaxial side of the primordium, whereas, in the abaxial tissues of the primordium, a default mechanism establishes ventral identity. The dorsal and ventral identities suppress and exclude each other, leading to the establishment of two sharply separated domains. At the lateral edge of the primordium, the interaction between the domains leads to outgrowth of the blade [4]. Therefore, genes responsible for adaxial-abaxial patterning in leaf development would play a key role in controlling LA. Rice varieties show large variation in tiller angle size, the genetic basis of which has been extensively investigated using quantitative trait loci (QTL) analysis. Several underlying genes, including *LAZY1*, *PROG1*, *DWARF4*, and *TAC1*, associated with tiller angle, have been cloned through map-based cloning [5]–[8]. Maize also exhibits tremendous natural variations with regard to LA. However, unlike that in rice, the map-based cloning of genes has not been effectively used in maize due to the unavailability of a complete genome sequence before 2009, the large genome size [10] and the highly repetitive sequence content [11]. To date, no genetic loci that control LA in maize have been identified.

Thus far, only 5 research groups have focused on LA in individual biparentally derived maize mapping populations used for QTL mapping [12]–[16]. Ku et al. [16] detected a major QTL, qLA2, for LA in the 2.08 region of chromosome 2 between umc1987 and phi090 by using 229 F_2:3_ families and 222 genetic map markers in 3 environments, accounting for 7.3% of phenotypic variation. Comparative genomics between rice and maize shows that the *OsTAC1* region on rice chromosome 9 is highly similar to a homologous maize gene in the qLA2 region. The *TAC1* gene on rice chromosome 9 was the first identified gene controlling tiller angle in rice [7]. In varieties with expanded plant architecture, *TAC1* acts as a positive regulator of the size of tiller angle. In varieties with compact plant architecture, a single nucleotide polymorphism (SNP) occurring at the 3′-splicing site of the 1.5-kb intron from “AGGA” to “GGGA” decreases the expression level of *tac1*, resulting in a compact plant architecture with a tiller angle close to zero. Further sequence verification of the mutation at the 3′-splicing site of the 1.5-kb intron revealed that the *tac1* mutation “GGGA” was present in 88 compact japonica rice accessions, and *TAC1* with “AGGA” in 21 wild rice accessions and 43 indica rice accessions, all with the expanded plant architecture, indicating that *tac1* had been extensively utilized in densely planted rice grown in high-latitude temperate areas and at high altitudes where japonica rice varieties are widely cultivated [7]. Therefore, the objectives of this study were to (1) isolate the maize leaf angle control 1 (*ZmTAC1*) gene in maize, (2) investigate if the maize *ZmTAC1* gene co-localizes with the previously identified QTL for LA between umc1987 and phi090, and (3) determine if the maize *ZmTAC1* gene has a similar function as the rice *TAC1* gene.

## Materials and Methods

### Plant material

**Figure 1:**
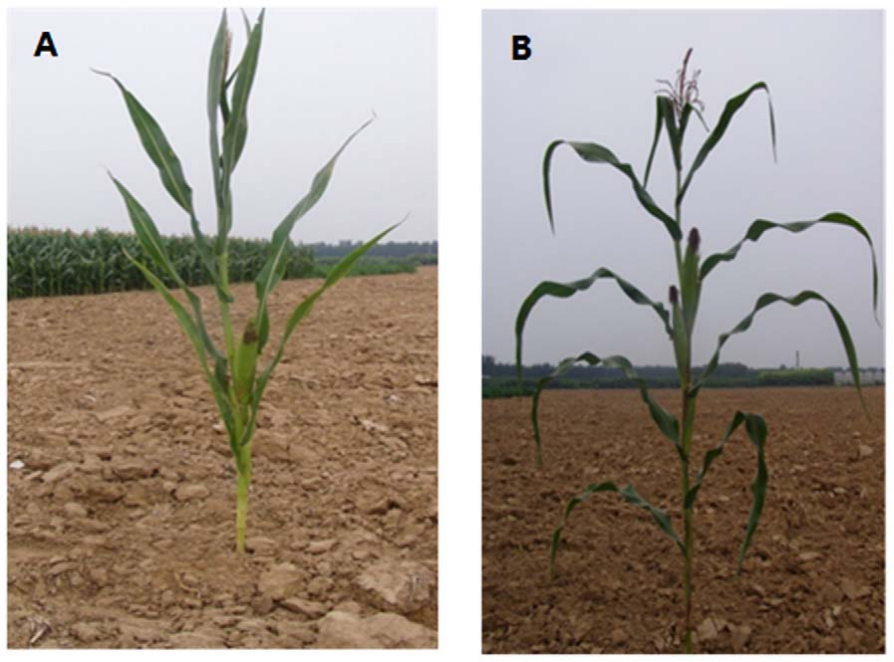
Phenotypes of Plant material.

The inbred line Yu82 with compact plant architecture derived from a Chinese Stiff Stalk germplasm and Shen137 with expanded plant architecture derived from a Chinese non-Stiff Stalk germplasm (Fig. 1) were used in this study to isolate the *ZmLAC1* sequence for molecular characterization analysis by comparing their dissimilarity using bioinformatics and patterns of gene expression. We used 18 inbred lines with expanded plant architecture and 13 inbred lines with compact plant architecture in this study for examining the dissimilarity in the 5′-UTR.

### Field trials and trait evaluation

The LAs (all leaves above the uppermost ear) from 31 inbred lines (Table 1) were evaluated in 2 environments: the same field Zhengzhou, Henan province, in 2009 and in 2010. The field experiment was followed by a randomized complete block design that was repeated 3 times. Each plot included 1 row that was 4 m long and 0.67 m wide, with a total of 15 plants at a density of 52,500 plants/ha. In order to study dynamic changes in LA, inbred lines Yu82 and Shen137 were planted in the plot including 6 rows at the same density. The LA was measured in both the beginning at the stage of the second leaf that fully expanded and the ending at the stage of the uppermost leaf in 25 plants of Yu82 and Shen137. The mean LA in 25 plants of Yu82 was compared with that in 25 plants of Shen137 at a similar stage of development. The definition of leaf angle was performed as described previously [16].

**Table 1:** The leaf angle measured at 10 days after pollen shed from 31 inbred lines.

Inbred line	Yu374	CML288	PH4CV	HZ4	Qi319	CML196	CML27	CML31	CML294	CML295	CML353	CML304	CML300	Zh425-322	CML301	CML312	CML242	Yu679	D321	P28	PH6WC	Ye478	Zh58	X9058	Huang C	Zong3	LD9801	CML338	Ji533	CML81	Tie7922
Leaf angle	33.73±1.23	23.50±1.17	28.07±1.32	27.33±1.30	31.77±1.26	30.40±1.32	41.07±1.05	50.03±1.04	58.67±1.26	67.13±1.27	68.97±0.97	69.75±1.24	50.4±1.31	49.63±1.28	49.83±1.21	46.08±1.19	64.17±1.01	34.76±0.96	13.55±1.03	9.39±0.87	17.94±1.24	13.33±1.12	11.61±1.22	11.57±0.98	17.05±1.24	18.57±1.03	22.09±1.13	22.3±1.08	21.09±1.24	22.43±1.07	17.95±1.00

### Genetic mapping and QTL mapping

The *TAC1* gene on chromosome 9 in rice was retrieved from the TIGR website (www.tigr.org) based on the cDNA sequence result reported by Yu et al. [7] and used to compare against 2 databases containing maize sequences (www.Maizegdb.org; www.ncbi.nlm.nih.gov). Primers were designed on the basis of the identified maize homologous sequences using Primer Premier 5.0 and used to screen for polymorphisms between the 2 maize inbred lines, Yu82 and Shen137, which had already been used to develop a set of F_2:3_ families to map QTL affecting LA [16]. Polymorphic primers were mapped onto the existing genetic map of the F_2_ population using Mapmaker/exp 3.0 [17]. QTL mapping in populations was carried out with the composite-interval mapping method of Windows QTL cartographer version2.5 software [18].

### DNA sequencing and identification of the genotype in the *cis*-element of 5′-UTR

A 2324-bp genomic fragment of *TAC1* gene was amplified from Yu82 and Shen137 using the primers 5′-CACTACTACCCCCGAGGA-3′ and 5′- TTCTTGGGTGATGTGTTA-3′. The polymerase chain reaction (PCR) products were purified and sequenced directly. The 488-bp fragment containing the *cis*-element of 5′-UTR was amplified to identify the genotypes in the *cis*-element of 5′-UTR from 31 inbred lines using the primers 5′-CACTACTACCCCCGAGGA-3′ and 5′- ATGCATCCTCCGATTCAA-3′.

### RNA analysis

In this study, each sample from leaf blades, leaf pulvinus, leaf sheath, SAM, and root from plants of Yu82 and Shen137 at the 3-leaf to 18-leaf stage was immediately frozen in liquid nitrogen and stored at −80°C prior to RNA extraction. For development-related qRT-PCR, total RNA of each tissue sample was extracted from three different, randomly selected plants. Three biological replicates were collected at the same time.

The first-strand cDNA was synthesized from total RNA and used as cloning templates for reverse transcriptase (RT)-PCR and real time-PCR. The primers 5′-CACTACTACCCCCGAGGA-3′ and 5′-AAGGAATCTGAGCCATAGGG-3′ were designed to amplify the full-length cDNA of *ZmTAC1* (GenBank accession No. GQ450293) using one-step RT-PCR. The amplified product was recovered and cloned into the pMD18-T vector (TaKaRa, Shanghai, China). The sequences of the RT-PCR primers were 5′-ATCCATGGTAGCAAAGCC-3′ and 5′-TCATTCCTGGGTTCCTCA-3′ for the *ZmTAC1* gene, and 5′-CCTGCGGCTTAATTGACTC-3′ and 5′- GTTAGCAGGCTGAGGTCTCG-3′ for the 18S gene, which served as control.

### Cleaved amplified polymorphism marker development

We noted a change in the position of the recognition site of the restriction enzyme *Bsm*I in the *ZmTAC1* sequences of Yu82 and Shen137. Therefore, a new primer pair was designed for this cleaved amplified polymorphism (CAP) marker (5′-CTGCCAGGTGTTTCATTG-3′ and 5′-AGGGCTTCAGTATCTTTCTC-3′). Then, the F_2_ population from the cross between Yu82 and Shen137 was genotyped using the CAP marker and QTL for LA were mapped using a set of 229 F_2:3_ families derived from the F_2_ population.

## Results

### Cloning of the *ZmTAC1* gene in maize

We created a list of candidate genes associated with LA from *Arabidopsis* and rice, and searched for their maize homologues by using BLAST-X at the National Center for Biotechnology Information (NCBI) website (http://www.ncbi.nlm.nih.gov/). DNA sequences originating from maize were considered to be homologous to candidate genes from other plant species if their BLAST-X homologies were smaller than E = 1.0×e^−15^. The second search method was BLAST-N directly on the MaizeGDB.org database utilizing the maize b73 reference bacterial artificial chromosome (BAC) library. The search results showed that the maize homologue of *OsTAC1* in rice was positioned in the qLA2 region between umc1987 and phi090 [16] by using the maize genome browser at MaizeGDB.org. Therefore, we designated the maize homologue of *OsTAC1* as *ZmTAC1*.

To identify if *ZmTAC1* was associated with LA, the genomic DNA and cDNA of *ZmTAC*1 were obtained from Yu82 and Shen137. Sequencing of the genomic DNA and cDNA of the *ZmTAC1* gene (Fig. S1) revealed that it contained 4 exons with 3 introns in its coding region, thereby producing a 792-nucleotide-long transcript encoding an unknown protein of 263 amino acids (Fig. S2), and a 286-bp fragment in the 5′-UTR and a 68-bp fragment in the 3′-UTR. The full-length cDNA sequence in coding region of *ZmTAC1* was identical to that of *ZmTAC1* reported by Yu et al [7]. *ZmTAC1* had high similarity with the corresponding gene in rice, indicating that the gene was conserved [7].

### Sequence difference of *ZmTAC1* in different inbred lines

Further comparison of the sequences of *ZmTAC1* from Yu82 and Shen137 revealed an important single-nucleotide mutation: “C” at the −28 bp site in 5′-UTR contained 286-bp sequence of *ZmTAC1* for Shen137 mutated into “T” at the same site for Yu82. The results from the Plantcare (http://bioinformatics.psb.ugent.be/webtools/plantcare) analysis revealed that “CTCC” in 5′-UTR contained 286-bp sequence of *ZmTAC1* was a cis-element recognized and bound by the CAG-motif, which was part of a light responsive element. The cis-element “CTCC” for Shen 137 became “CTCT” in 5′-UTR for Yu82 as a result of the single nucleotide mutation at the 28 bp site. This change led to a shift of cis-element “CTCC” 2-bp backward for Yu82 corresponding to the location for Shen137. 3-bp “CTC” for Shen 137 but only 1-bp “C” for Yu82 in cis-element “CTCC” could be bound by the sequence of the CAG-motif (Fig. S3). This findings suggested the different locations of cis-element “CTCC” in 5′-UTR might influence the binding capability of the CAG-motif. In addition, the predicted amino acid sequences encoded by *ZmTAC1* and *Zmtac1* remained unchanged although a nonsense change occurred in the second exon (Fig. S1). None of the different nucleotides in the promoter region of Yu82 and Shen137 could be linked to the expanded and compact architecture.

To investigate any possible correlation between compact plant architecture and the *cis*-element from “CTCC,” sequence verification was performed on 31 maize inbred lines comprising 18 expanded inbred lines and 13 compact inbred lines (Table 1). The results showed that *ZmTAC1* with “CTCC” was present in 13 compact inbred lines that their LAs ranged from 9.39° to 22.43°, and *ZmTAC1* with “CCCC” was present in 18 expanded inbred lines that their LAs ranged from23.50° to 69.75°, indicating that “CTCC” at the −28 bp mutation site in 5′-UTR of *ZmTAC1* was always associated with compact plant architecture, whereas “CCCC” was always associated with all the expanded plant architecture (Table 1).

### *ZmTAC1* expression pattern

**Figure 2:**
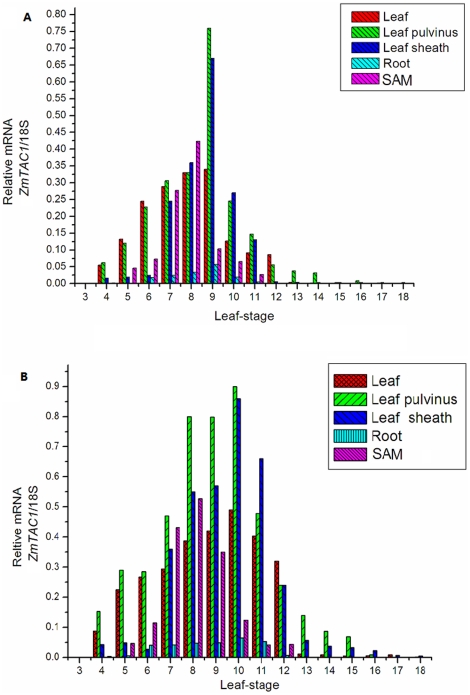
The expression of *ZmTAC1* at different tissues in Yu82 and Shen137.

**Figure 3:**
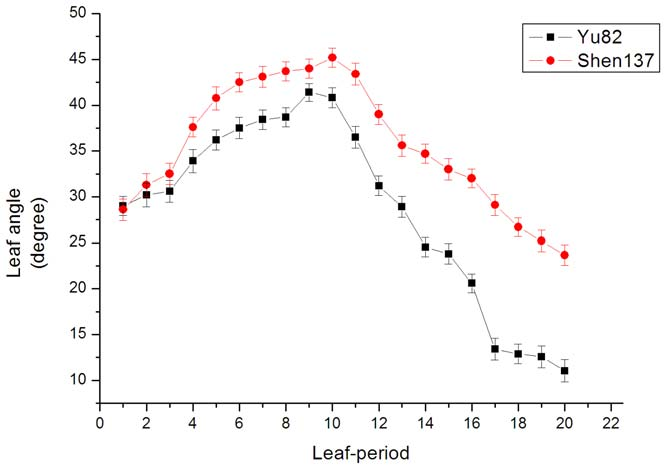
The LA change over developmental stage.

Real-time PCR was performed to determine the expression pattern of *ZmTAC1*. As shown in Fig. 2, the expression was significant in the leaf blade, leaf sheath, leaf pulvinus, and SAM in plants at the 3-leaf to 13-leaf stage, but not significant in the root. In addition, the expression was the highest in the leaf-sheath pulvinus, less in the leaf and SAM, and the lowest in the root at the 9-leaf to 10-leaf stage when peak expression occurred over developmental stages. As shown in Fig. 3, the value of leaf angle was higher in Shen137 than that in Yu82 through developmental stages, and the maximum difference between two lines is achieved from 17-leaf to 20-leaf stages despite consistent tendency to change over developmental stages for both Yu82 and Shen137 strains. In both cases, the LAs were small at the 2-leaf to 3-leaf stage, gradually increased at the 4-leaf stage, were the largest at the 10-leaf stage, and decreased after the 11-leaf stage.

**Figure 4:**
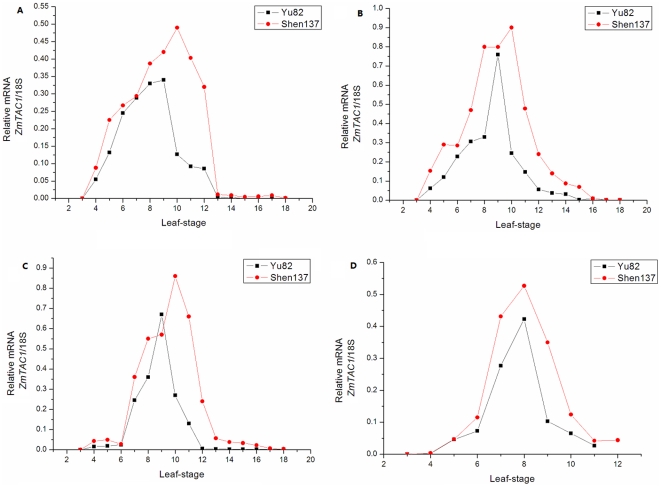
The expression of *ZmTAC1* at different stages in Yu82 and Shen137.

Real-time PCR analysis showed that the mRNA expression of *ZmTAC1* was higher in Shen137 than that in Yu82 in all developmental stages and different tissues, except that the expression level of leaf sheath at 9-leaf stage were higher in Yu82 than that in Shen137 (Fig. 4). In addition, expression patterns present in different developmental stages were found. The mRNA accumulation of *ZmTAC1* in leaf blade was low at the 2-leaf to 3-leaf stage, increasing at the 4-leaf stage, reaching a peak at the 10-leaf stage, and decreasing to minimum from the 11-leaf stage to 13-leaf stage for both of Yu82 and Shen137 (Fig. 4). The expression patterns in the leaf sheath and leaf pulvinus were similar to that in the leaf blade before the 13-leaf stage, but *ZmTAC1* was slightly expressed in the leaf sheath and leaf pulvinus in Shen137 and not in Yu82 after the 13-leaf stage. *ZmTAC1* exhibited peak expression in the SAM at the 8-leaf stage in Yu82 and Shen137 because tassels emerged at the 11-leaf and 12-leaf stage, respectively. Comparison of the changes in leaf angle and in mRNA expression of *ZmTAC1* over developmental stages suggested that the change in *ZmTAC1* expression might contribute to leaf angle polymorphism.

### *ZmTAC1* preliminary mapping

We noted a change in the position of the recognition site of the restriction enzyme *Bsm*I in the *ZmTAC1* sequences of Yu82 and Shen137. Therefore, a new CAP marker was developed. Next, the F_2_ population from the cross between Yu82 and Shen137 was genotyped by using the CAP marker, and QTL for LA were mapped by using a set of 229 F_2:3_ families derived from the F_2_ population [16]. The results showed that the CAP marker representing *ZmTAC1* could be mapped to the QTL qLA2 region on chromosome 2 between umc1987 and phi090 (Fig. S4). The mapping result was identical with the location on the physical map of sequenced and ordered BAC clones using the maize genome browser at MaizeGDB.org. The qLA2 explained the 7.3% of the phenotypic variation. The results showed that *ZmTAC1* is likely to be encoded in the qLA2 region.

## Discussion

Maize plant architecture is considered as one of the most important agronomic traits and has long attracted the attention of breeders for achieving the ideal plant architecture to improve grain yield. Plant architecture determines planting density, further largely influences photosynthetic efficiency, disease resistance and lodging resistance. One of our interests was to investigate the genetic controls underlying LA for improving maize plant architecture. In this study, we cloned the maize ortholog, *ZmTAC1* gene, of the *OsTAC1* in rice. We further confirmed the *ZmTAC1* potential function regulating the leaf angle in maize through the three approaches: a) sequence dissimilarity analysis by using 31 defferent maize inbred lines from temperate and tropic regions, b) consistent QTL mapping results by using QTL qLA2 from a set of 229 F2:3 families and by the functional CAP marker developed from *ZmTAC1* sequence, and c) consistent changes between *ZmTAC1* expression and LA polymorphism in both lines and developmental stages. These three consistent findings indicated that *ZmTAC1* might play an important role in the leaf regulatory pathway.

### The change from “CCCC” to “CTCC” in the 5′-UTR of the *ZmTAC1* locus influencing the size of LA

Further comparison of the sequences of *ZmTAC1* from Yu82 and Shen137 revealed the important single nucleotide mutation of cis-element in 5′-UTR could influence the binding capability of the CAG-motif, and thus expression level of *pZmTAC1*. Sequence verification performed on 31 maize inbred lines (Table 1) showed that the single nucleotide mutation in the 5′-UTR of the *ZmTAC1* locus might be associated with the size of LA and “CTCC” at the −28 bp site in 5′-UTR could resulted in a compact plant architecture with erect angles in maize.The mutation in the *ZmTAC1* locus in maize was similar to that in the *OsTAC1* locus in rice verified by Yu et al. [7] despite difference between at the 5′-UTR in the *ZmTAC1* and at the 3′-UTR in rice. Yu et al. identified the single nucleotide mutation at 3′-UTR of *OsTAC1* in rice led to a compact plant architecture with erect tillers based on sequence verification on 152 rice accessions comprising 88 compact japonica cultivators and 54 types of rice of the expanded form. Their results showed that the *tac1* mutation “GGGA” was always associated with compact plant architecture, whereas all expanded forms harbored the *OsTAC1* gene (“AGGA”) [7].

### *ZmTAC1* is involved in the mRNA level and leaf development in maize

The 5′-UTR of many genes has been shown to be essential for the regulation of gene expression at transcriptional levels [19], [20]. To verify the effect of the *ZmTAC1* 5′-UTR on the regulation of gene expression, we investigated the expression patterns of *ZmTAC1* from Yu82 and Shen137 in different tissues at different developmental stages of maize plants. The results showed some discrepancy in *ZmTAC1* mRNA accumulation in different tissues and in different developmental stages of Yu82 and Shen137. Leaf tissues might directly affect plant architecture development and sustainability [8], [9]. Our results showed that significant accumulation of *ZmTAC1* mRNA appeared in leaf tissues (the leaf pulvinus, leaf sheath, leaf blade) and SAM, but not much in the roots in both of Yu82 and Shen137. However, accumulation of *ZmTAC1* mRNA in different tissues was higher in Shen137 than that in Yu82. The *ZmTAC1* expression pattern of different tissues in maize were consistent with the *OsTAC1* expression pattern in rice [7].Yu et al. [7] showed significant accumulation of *OsTAC1* mRNA in the tiller base, tiller node, and sheath pulvinus in rice.

Comparison of the changes in leaf angle and in mRNA expression of *ZmTAC1* over developmental stages in Yu82 and Shen137 showed that the mRNA expression of *ZmTAC1* was higher in Shen137 than that in Yu82, and that the expression peak of *ZmTAC1* in leaf tissues at 10-stage was consistent with the leaf angle maxima at the same stage (Figs. 3 and 4). The findings suggested that the *ZmTAC1* expression level was positively correlated with leaf angle size, and *ZmTAC1* might contribute to leaf angle polymorphism by varying mRNA level regulated jointly by the 5′-UTR.

In regard with Yu82 and Shen137, the maximum difference in leaf angle appeared at late developmental stages, by which period the *ZmTAC1* expression levels were down-regulated to nearly zero, and their greatest expression difference occurred in the middle developmental stage, at which time their leaf angle difference is rather small. The results showed that *ZmTAC1* mRNA quantity demanded was on the rise with the increase of developing leaf number at early developmental stages for Yu82 and Shen137, and that *ZmTAC1* expresssion reached peak and leaf angles of parents were the largest at the 10-leaf stage. With accomplishing of lower leaves and increase of upper developing leaf number, *ZmTAC1* mRNA expression and leaf angles decreased from the 11-leaf stage. However, comparisons across two inbred lines found the rate of decrease of *ZmTAC1* mRNA expression in Shen137 was smaller than in Yu82, and expressive disparity in the leaf blade, leaf sheath, leaf pulvinus in Shen137 preserved into 13, 16, and 17 leaf-stages, respectively. Therefore, *ZmTAC1* mRNA quantity demanded in tissue development of upper leaf could be lower than in tissue development of middle leaf. The regulatory mechanism of *ZmTAC1* in maize was similar to that of *OsTAC1* in rice. It could be the effect of other genes associated with leaf angle influencing variation of LA and/or the repression of endogenous *ZmTAC1* by hitherto unidentified factor(s) in response to the developmental signal in leave tissues.

### Further function verification for *ZmTAC1*

The gene function can be indirectly validated by QTL mapping by functional CAP marker. In this study, we developed a functional CAP marker based on the sequence difference between Yu82 and Shen137 to further verify the function of *ZmTAC1*. The results showed that the *ZmTAC1* was found to be located in the region of qLA2 by using the developed CAP marker and the F_2:3_ families from the cross between Yu82 and Shen137 (Fig. S4). The findings suggested *ZmTAC1* might be a candidate gene in the qLA2 region. Therefore, the *ZmTAC1* has a potential function in regulating the leaf angle of maize. In addition, such a functional marker is a good molecular marker for the LA and can be applied to marker-assisted selection to modify crop architecture in maize breeding.

Functional studies of *ZmTAC1* can be directly proven utilizing transgenic maize. So our functional complementation experiment is under way in our lab. The results could provide the important information to further study and applicate to maize breeding even if functional complementation experiment has not completed.

Maize with desirable plant architecture can be created by improving plants in modern crop breeding programs. In this study, we proved that *ZmTAC1* is essential for the regulation of LA, and the molecular characterization of *ZmTAC1* not only shed light on leaf development but also provided an opportunity to optimize crop plant architecture by molecular design and to improve grain yield in future crop breeding.

## Supporting Information

Figure S1 **The comparison of sequence for** ***ZmTAC1*** **in Yu82 and Shen137.**
(TIF)

Figure S2 **The amino acid sequence of** ***ZmTAC1*** **.**
(TIF)

Figure S3 **The sequence comparison of cis-element in 5′-UTR of** ***ZmTAC1*** **for Yu82 and Shen137.**
(TIF)

Figure S4 **The location of** ***ZmTAC1*** **on chromosome 2.**
(TIF)
