# Supplementary figures and images for: Cloning and Characterization of a Putative TAC1 Ortholog Associated with Leaf Angle in Maize (Zea mays L.)
Source: PLoS One. 2011 Jun 7;6(6):e20621. doi: 10.1371/journal.pone.0020621 (PMC3110200; doi:10.1371/journal.pone.0020621)

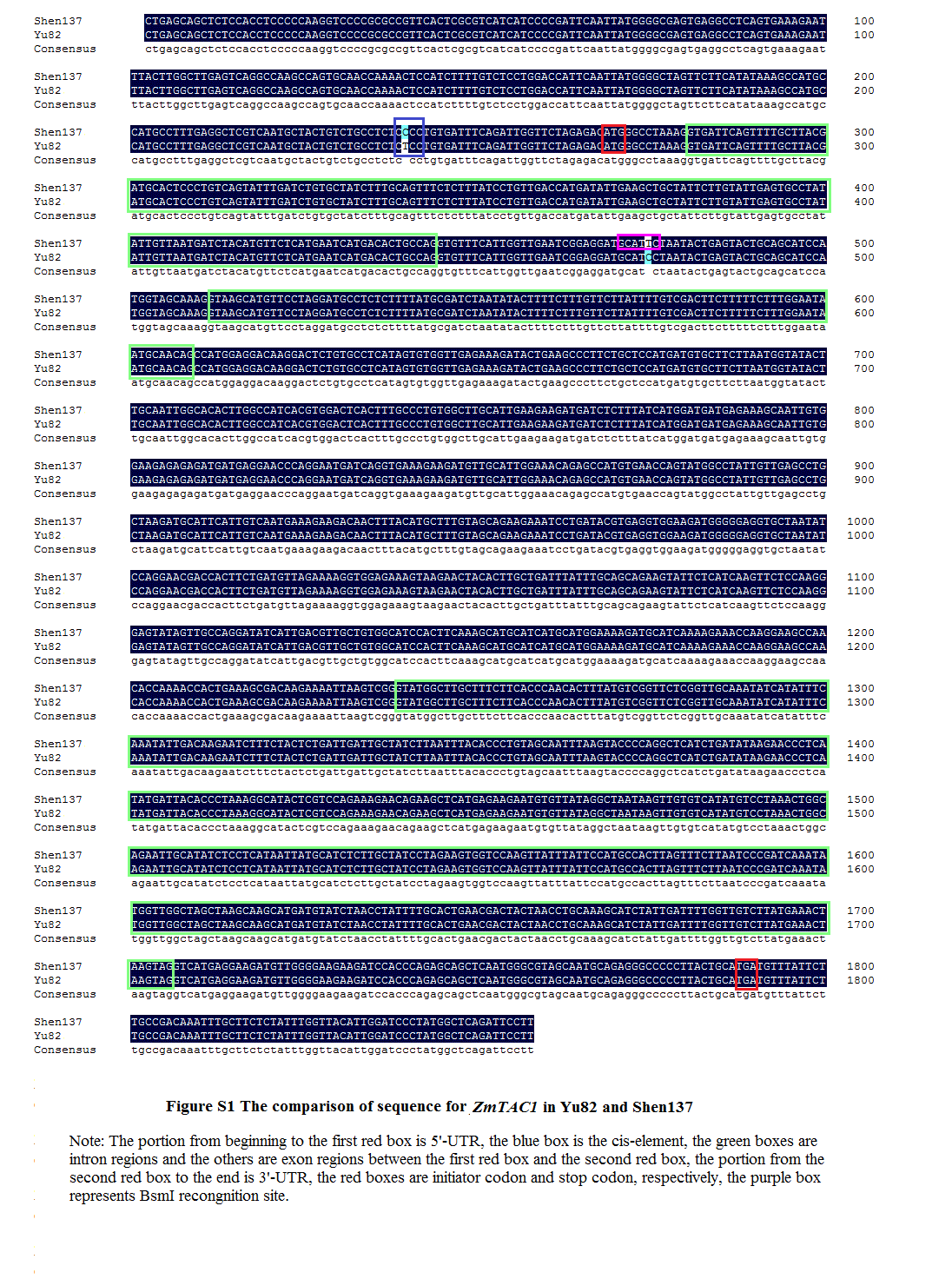

Supplement: Figure S1 — The comparison of sequence for ZmTAC1 in Yu82 and Shen137. (TIF) [file pone.0020621.s001.tif]

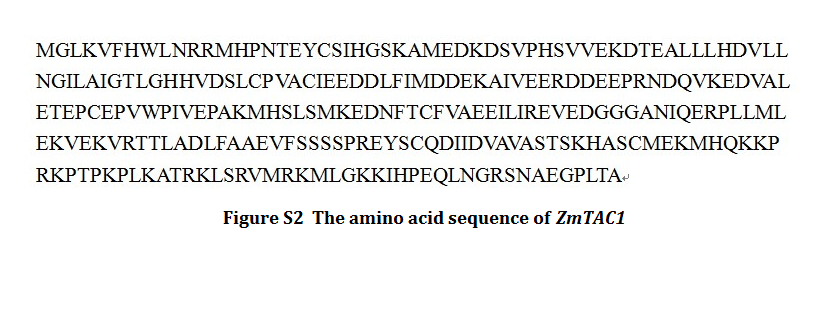

Supplement: Figure S2 — The amino acid sequence of ZmTAC1 . (TIF) [file pone.0020621.s002.tif]

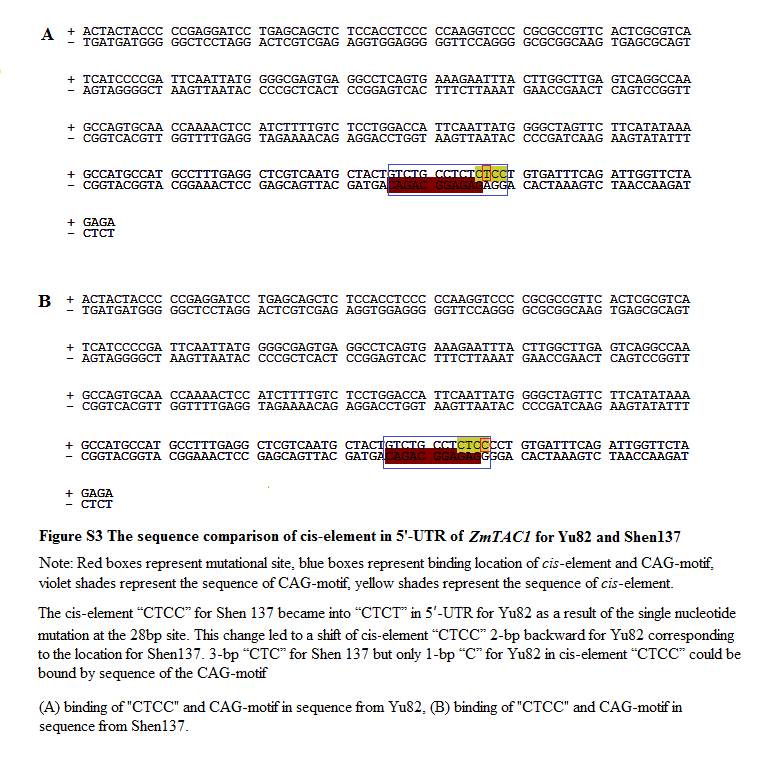

Supplement: Figure S3 — The sequence comparison of cis-element in 5′-UTR of ZmTAC1 for Yu82 and Shen137. (TIF) [file pone.0020621.s003.tif]

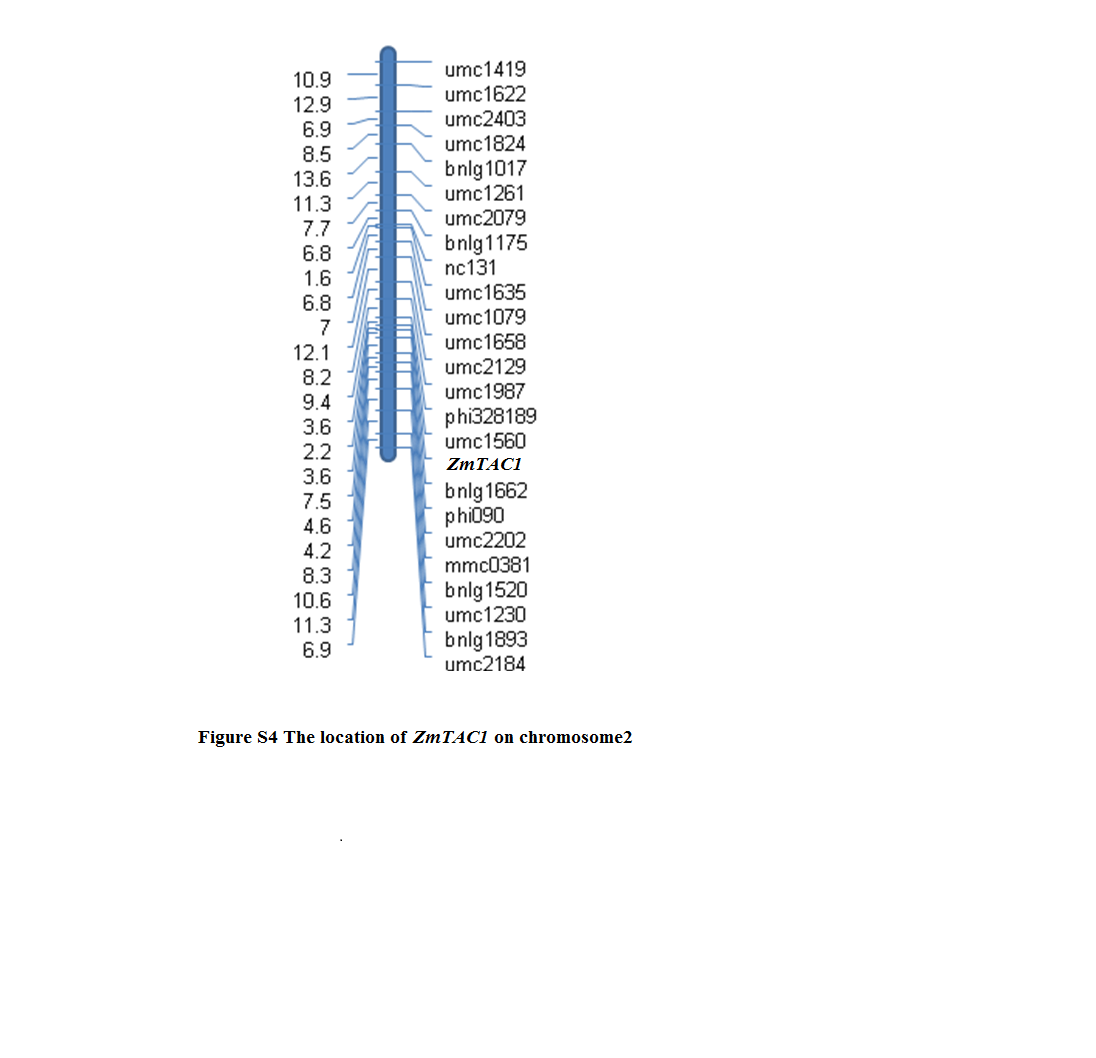

Supplement: Figure S4 — The location of ZmTAC1 on chromosome 2. (TIF) [file pone.0020621.s004.tif]
